# The effects of spatial structure, frequency dependence and resistance evolution on the dynamics of toxin-mediated microbial invasions

**DOI:** 10.1111/eva.12284

**Published:** 2015-07-16

**Authors:** Ben Libberton, Malcolm J Horsburgh, Michael A Brockhurst

**Affiliations:** 1 Department of Integrative Biology, University of LiverpoolLiverpool, UK; 2 Department of Biology, University of YorkYork, UK; 3 Karolinska InstituteSE-171 77, Stockholm, Sweden

**Keywords:** community ecology, experimental evolution, interference competition, invasion, spatial structure, staphylococci, toxin production

## Abstract

Recent evidence suggests that interference competition between bacteria shapes the distribution of the opportunistic pathogen *Staphylococcus aureus* in the lower nasal airway of humans, either by preventing colonization or by driving displacement. This competition within the nasal microbial community would add to known host factors that affect colonization. We tested the role of toxin-mediated interference competition in both structured and unstructured environments, by culturing *S. aureus* with toxin-producing or nonproducing *Staphylococcus epidermidis* nasal isolates. Toxin-producing *S. epidermidis* invaded *S. aureus* populations more successfully than nonproducers, and invasion was promoted by spatial structure. Complete displacement of *S. aureus* was prevented by the evolution of toxin resistance. Conversely, toxin-producing *S. epidermidis* restricted *S. aureus* invasion. Invasion of toxin-producing *S. epidermidis* populations by *S. aureus* resulted from the evolution of toxin resistance, which was favoured by high initial frequency and low spatial structure. Enhanced toxin production also evolved in some invading populations of *S. epidermidis*. Toxin production therefore promoted invasion by, and constrained invasion into, populations of producers. Spatial structure enhanced both of these invasion effects. Our findings suggest that manipulation of the nasal microbial community could be used to limit colonization by *S. aureus,* which might limit transmission and infection rates.

## Introduction

*Staphylococcus aureus* colonizes the lower portion of the nasal airway (anterior nares) persistently in around 20% of the human population (Van Belkum et al. 2009). Although persistent nasal colonization by *S. aureus* (carriage) is typically asymptomatic, it is a risk factor for infection in specific patient groups (Von Eiff et al. 2001). These infections can be recurrent and respond poorly to treatment (Kreisel et al. 2006), while the risk of infection is significantly higher for immunocompromised carriers, with increased severity and mortality rates (Yu et al. 1986; Hoen et al. 1995; Senthilkumar et al. 2001).

Studies have revealed many diverse host, bacterial and environmental factors that influence *S. aureus* carriage. Host factors include genetic variation of the immune response (Van den Akker et al. 2006; Ruimy et al. 2010) and being part of certain patient groups give higher rates of carriage (Atela et al. 1997; Lederer et al. 2007). *S. aureus* determinants that affect carriage include secreted components associated with immune system interaction (De Haas et al. 2004; Genestier et al. 2005; Rooijakkers et al. 2005) or components of the bacterial cell surface (Kreikemeyer et al. 2002; Clarke et al. 2004; Heilmann et al. 2004).

The nasal microbial community is mainly comprised of *Corynebacterium*,*Propionibacterium* and *Staphylococcus*, with the latter genus constituting between 15% and 60% of the nasal microbial community and mainly comprising the species *S. aureus* and *Staphylococcus epidermidis* (Wos-Oxley et al. 2010). There is increasing evidence that the nasal microbial community may contribute to determining *S. aureus* carriage (Peacock et al. 2001; Frank et al. 2010; Wos-Oxley et al. 2010; Yan et al. 2013; Libberton et al. 2014). One well-described staphylococcal mechanism is via competition arising from allelic variation within *agr*-dependent signal transduction (Regassa et al. 1992; Yarwood et al. 2002; Weinrick et al. 2004; Schlievert et al. 2007; Horswilll and Nauseef 2008; Peterson et al. 2008). Several studies report negatively associated distributions of *S. epidermidis* and *S. aureus* across nasal communities, suggesting that these species engage in one-way or mutual exclusion (Lina et al. 2003; Frank et al. 2010; Wos-Oxley et al. 2010; Libberton et al. 2014). Several potential biochemical mechanisms for these observed patterns have been suggested. Iwase et al. (2010) identified that *S. epidermidis* can displace *S. aureus* from the nasal niche by serine protease-mediated biofilm disruption; Lina et al. (2003) showed that quorum sensing interference could contribute to competition whereby different agr types of *S. aureus* and *S. epidermidis* could not inhabit the same community. In addition, *S. aureus* and *S. epidermidis* both secrete a variety of toxins, which can kill interspecific competitors (Nascimento et al. 2012; Sandiford and Upton 2012; Peschel and Otto 2013)

Here, we constructed simple *in vitro* communities of *S. epidermidis* and *S. aureus* to explore the hypothesis that toxin-mediated killing of competitor species (interference competition) could contribute to the observed negatively associated distributions of these species in nasal communities. Theory predicts that interference competition can both promote and prevent invasion of resident communities. Invasion is promoted when invading populations produce toxin(s) that can kill the resident. However, the cost of producing toxins must be lower than the benefits gained from producing them, and the benefits must not be shared between invader and resident populations. If these criteria are not met, then the interference competition will reduce the chance of invasion (Chao and Levin 1981). Resident populations that produce toxins have been shown to restrict invasion by toxin-sensitive populations (Adams et al. 1979; Chao and Levin 1981; Durrett and Levin 1994; Frank 1994; Duyck et al. 2006; Allstadt et al. 2012). We explored two scenarios in which toxin production by *S. epidermidis* could drive exclusion of *S. aureus*: first, where resident toxin-producing *S. epidermidis* prevent invasion by susceptible *S. aureus,* and second, where invading toxin-producing *S. epidermidis* displace a resident susceptible *S. aureus* population. In addition, we manipulated two ecological parameters that influence the success of toxin-mediated interference competition, specifically, the spatial structure of the environment and the starting frequency of invaders.

In bacteria, interference competition is typically mediated by environmentally secreted toxins, and therefore, it is likely to be affected by environmental spatial structure. Experiments with *Escherichia coli* have demonstrated that in spatially structured environments (agar plates), bacteriocin producers invaded from very low starting frequency (0.001) into bacteriocin-sensitive populations. By contrast, in the absence of spatial structure (shaken liquid broth), much higher initial frequencies of producers (0.1) were required for successful invasion (Chao and Levin 1981). Spatially structured environments were proposed to promote invasion of toxin producers because clustering of producers enables toxins to reach higher local concentrations (Majeed et al. 2011). As such, the benefits of costly toxin production can accrue to small founding populations. By contrast, in spatially unstructured environments, rapid diffusion of the bacteriocin and quorum sensing molecules away from producing cells of *E. coli* required bacteriocin producers to exceed a higher threshold frequency before the benefits of bacteriocin production could be realized (Chao and Levin 1981; Tait and Sutherland 2002; Greig and Travisano 2004). Similar frequency-dependent invasion effects of toxin producers were demonstrated in spatially structured populations of the yeast *Saccharomyces cerevisiae* (Greig and Travisano 2004). We predicted therefore that toxin-producing *S. epidermidis* strains would be better able to invade-from-rare than nonproducing strains and would do so from lower starting frequencies in more highly spatially structured populations.

Ecological theory proposes that interference competition by a resident species should prevent invasion by a susceptible species irrespective of spatial structure (Adams and Traniello 1981; Doyle et al. 2003). When a toxin kills susceptible immigrants, invaders are unable to sustain a viable population; in population ecology, such hostile environmental patches are often termed black hole sinks (Holt and Gaines 1992). Evolutionary theory also proposes that there is potential for a susceptible invading population to evolve resistance to a toxin and that the probability of this will depend upon the frequency of invaders and the spatial structure of the environment (Chao and Levin 1981; Holt et al. 2003). Several theoretical models predict that the likelihood of adaptation to a black hole sink environment increases with the frequency of immigrants from the source population (Gomulkiewicz et al. 1999; Holt et al. 2003). Higher immigration rates will increase the probability that immigrants carry beneficial mutations that are pre-adapted to survive the conditions of the black hole sink (Holt and Gaines 1992; Perron et al. 2008). Therefore, invading *S. aureus* populations are more likely to contain mutants resistant to *S. epidermidis* toxins when invading from higher starting frequencies. However, the spread of these beneficial resistance mutations is likely to be impeded in more highly spatially structured environments. This is because competition of the beneficial mutant can only occur at the edge of a colony, and as the colony grows, a smaller proportion of the mutant population will be competing with the ancestral genotype (Habets et al. 2007). Taken together, we predict therefore that nonproducing residents will be more easily invaded, that resistance of the invader to inhibitory toxins is more likely to evolve when invaders are at a high starting frequency and that resistant mutants that evolve will be more likely to invade in unstructured environments.

To test these predictions, we performed competition experiments whereby toxin-producing and nonproducing nasal isolates of *S. epidermidis* were invaded from three starting frequencies (0.1, 0.01 and 0.001) into resident populations of toxin-sensitive *S. aureus*. Conversely, to test whether *S. aureus* invasion could be restricted by *S. epidermidis* toxin production, we performed the reciprocal invasion of *S. aureus* from three starting frequencies (0.1, 0.01 and 0.001) into resident populations of toxin-producing and nonproducing *S. epidermidis*. All competitions were propagated for 7 days on solid agar with daily transfer of communities to fresh medium; in half of the replicates, population structure was maintained at each transfer, whereas in the other half of the replicates, the population structure was homogenized at each transfer.

## Materials and methods

### Culture conditions

All bacterial strains used in this study were cultured at 37°C in 10 mL BHI broth shaken at 200 rpm and on agar-solidified BHI medium (brain–heart infusion solids (porcine), 17.5 g/L; tryptose, 10.0 g/L; glucose, 2.0 g/L; sodium chloride, 5.0 g/L; disodium hydrogen phosphate, 2.5 g/L) (Lab M, Heywood, UK). Chemicals were obtained from Sigma-Aldrich Co., UK.

### Selection of nasal isolates

Four independent *S. epidermidis* isolates were selected from a previous study that sampled the anterior nares of 60 healthy volunteers (Libberton et al. 2014): two isolates were toxin producers as revealed in a deferred inhibition assay by their killing of *S. aureus* [zone of clearing when a lawn of *S. aureus* strain SH1000 was sprayed over them (Nascimento et al. 2012)]; two isolates were toxin nonproducers based on not reducing viability of strain SH1000. SH1000 displayed no growth inhibition activity against any of the selected *S. epidermidis* strains in the deferred inhibition assay (Nascimento et al. 2012). Of the two toxin-producing *S. epidermidis* strains, B180 produced an inhibition area that was around ten times greater than that of B155. We first established that the *S. epidermidis* strains had comparable growth rates to SH1000. An overnight culture of each strain (Table1) was inoculated (1% inoculum) into 200 *μ*L of BHI broth in a 96-well plate. The 96-well plates were incubated at 37°C for 8 h, and OD_600_ readings were taken at 20-min intervals. The doubling time (min) was then calculated (Table2) using the following formula where *T*_d_ is the doubling time; *t*_1_ and *t*_2_ are two consecutive time points throughout the bacterial growth; and *d*_1_ and *d*_2_ are the corresponding OD_600_ readings at *t*_1_ and *t*_2_. 
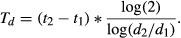


**Table 1:** Strains used in this study

Species	Strain identification	Reference
*S. aureus*	SH1000	Horsburgh et al. (2002)
*S. epidermidis*	B155 (inhibitor producing)	Libberton et al. (2014)
*S. epidermidis*	B180 (inhibitor producing)	Libberton et al. (2014)
*S. epidermidis*	B035 (noninhibitor producing)	Libberton et al. (2014)
*S. epidermidis*	B115 (noninhibitor producing)	Libberton et al. (2014)

**Table 2:** Doubling times of strains used in this study. The doubling times in minutes were compared to SH1000 (*S. aureus*) as a control using a *post hoc* Dunnett’s test. There is no significant difference between any of the *S. epidermidis* strains tested and the *S. aureus* strain SH1000 used in this study

	Doubling time (min)	*T*-value	*P*-value
SH1000	116.45	NA	NA
B180	116.06	−0.074	1.0000
B155	110.11	−1.203	0.5689
B115	120.49	0.767	0.8579
B035	123.34	1.307	0.4970

### Competition experiments

All strains were cultured on BHI agar plates prior to competition experiments. Bacteria were cultured for 18 h on 50-mm-diameter BHI agar plates, and the lawns of *S. aureus* (SH1000) and *S. epidermidis* strains (resident and invader – Table1) were then scraped off the agar plates and suspended in 10 mL of PBS by vortexing thoroughly. The cfu/mL in each tube was equalized by diluting the cell suspensions in PBS and comparing the OD_600_ of each suspension (approximately 5 × 10^8^ cfu/mL for *S. aureus* and *S. epidermidis*, determined by viable count). Both species were then mixed together in a final volume of 10 mL PBS, with the invader at different frequencies (ratios) to the resident (0.1:1, 0.01:1, 0.001:1). For brevity, these ratios are referred to in this manuscript as frequencies, and only the first number in the ratio pair is used to define each frequency. The mixtures were vortexed thoroughly before 50 *μ*L (containing approximately 2.5 × 10^6^ cells) was plated onto 25 mL BHI agar and incubated at 37°C. Six replicate communities (structured and unstructured, in triplicate) were established at each starting frequency. The communities were transferred to a new agar plate every day for 7 days. Half of the replicates underwent a regime whereby the transfers were made by replica plating with velvet (Lederberg and Lederberg 1952) to maintain spatial structure. While the other half of replicates underwent a mixed regime whereby the spatial structure was destroyed every 24 h transfer by scraping the entire bacterial lawn off the plate and transferring to 10 mL of sterile PBS, before thoroughly vortexing and pipetting 50 *μ*L onto a new plate to complete the transfer. Each set was performed in triplicate. Viable counts for each isolate were calculated every second day. On the structured plates, this was achieved after replica plating from viable counts of the remaining lawn; colonies were differentiated by colony morphology and pigmentation. *S. aureus* SH1000 possesses a distinct yellow carotenoid pigment which was stable over the course of these experiments. Raw data for the experiments are presented in appendices (Figs 7 and 8).

### Deferred inhibition spray assay

A deferred inhibition spray assay was performed to determine whether *S. aureus* clones had developed resistance to the toxin-producing *S. epidermidis* strains. The assay was performed on 10 clones from each experiment. A 25-*μ*L spot (approximately 10^8^ cells) of an overnight bacterial culture was pipetted onto the centre of an agar plate containing 15 mL of BHI agar (Lab M). The plates were incubated for 18 h at 37°C before 250 *μ*L of a 10-fold diluted overnight culture of a different strain (10^6^ cfu) was sprayed over the plate. The plates were incubated for a further 18 h after when the size of the inhibition zones produced by the central spot on the overlaid strain was assessed. The clarity of the inhibition zone was scored based on a simple scoring system of 1–4, 4 being completely clear and 1 being no detectable zone. The areas of any detectable zones were also recorded by measuring the diameter of the inhibition zone and the central colony.

### Data analysis

To quantify the success of the invasion, we calculated the selection rate constant for each invader using relative bacterial frequencies from day 0 and day 7 with the following equation.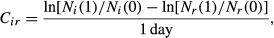 where *N*_*i*_ (0) and *N*_*r*_ (0) represent the initial densities of the competing populations *i* (invader) and *r* (resident), and *N*_*i*_ (1) and *N*_*r*_ (1) represent their densities after 1 day (Travisano & Lenski, 1996).

Negative values indicated that invasion was not possible, whereas positive values indicated invasion was possible. The invasion time-course data were visualized using plots of the natural log of the invader to resident ratio over time; selection rate constants were analysed in a three-way anova.

## Results

### Spatial structure promotes invasion by inhibitor-producing *S. epidermidis*

**Figure 1:**
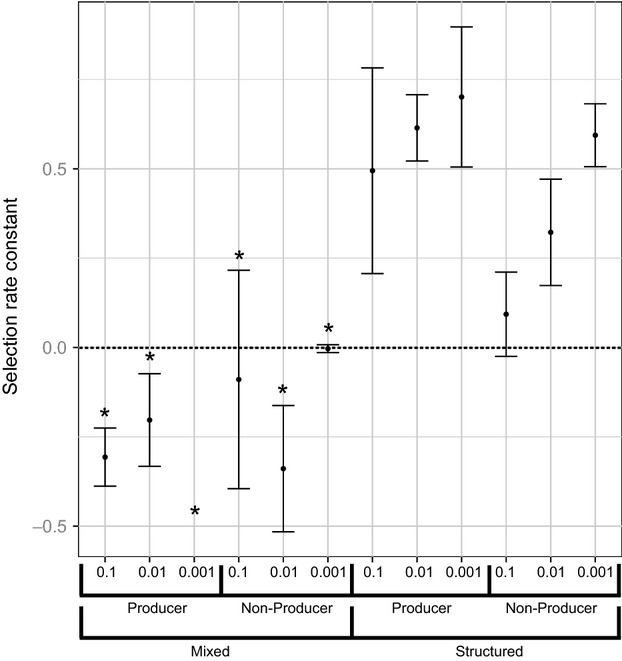
Selection rate coefficients for *Staphylococcus epidermidis* invading populations of *S. aureus* (SH1000). Toxin-producing *S. epidermidis* isolates (155 and 180) and nonproducing isolates (035 and 115) were invaded into populations of *S. aureus* (SH1000) at relative frequencies of 10, 100 and 1000. Each of the invasions was also carried out under a spatially structured treatment and a mixed treatment. Asterisks mark negative selection rate coefficients where invasion did not occur. Error bars represent the standard error of the mean.

Environmental structure promoted *S. epidermidis* invasion (structure, *F*_1,64_ = 322.77, *P* < 0.001) (Fig.1 and Table3), and this effect was stronger for *S. epidermidis* toxin producers than for nonproducers (structure × inhibition, *F*_1,64_ = 14.29, *P* < 0.001) (Table3). *S. epidermidis* was never able to successfully invade under mixed conditions (Fig.1 and Table3). However, *S. epidermidis* was more likely to persist at low frequencies and avoid extinction in mixed environments when initiated at a higher starting frequency (frequency × structure, *F*_1,64_ = 13.55, *P* < 0.001).

**Table 3:** Analysis of variance testing the main effects of successful invasion of *S. epidermidis* into populations of *S. aureus*. The table shows the results of a multifactorial anova. Both main effects and interactions are shown

	df	Sum sq	Mean sq	*F* value	*P* value
Frequency	1	0.1382	0.1382	3.4920	0.0662444
Structure	1	12.7710	12.7710	322.7662	<2.2e-16***
Inhibition	1	0.1452	0.1452	3.6690	0.0599020
Frequency × Structure	1	0.5359	0.5359	13.5452	0.0004798***
Frequency × Inhibition	1	0.0243	0.0243	0.6141	0.4361327
Structure × Inhibition	1	0.5652	0.5652	14.2852	0.0003474***
Frequency × Structure × Inhibition	1	0.0746	0.0746	1.8858	0.1744717

df, Degrees of freedom; Sum sq, sum of squares; Mean sq, Mean of squares; *F* value, *F* statistic for terms in the row; *P* value, significance.

Asterisks indicate the significance levels at different thresholds. ^***^P < 0.001.

### Invasion was impeded by evolution of resistance

**Figure 2:**
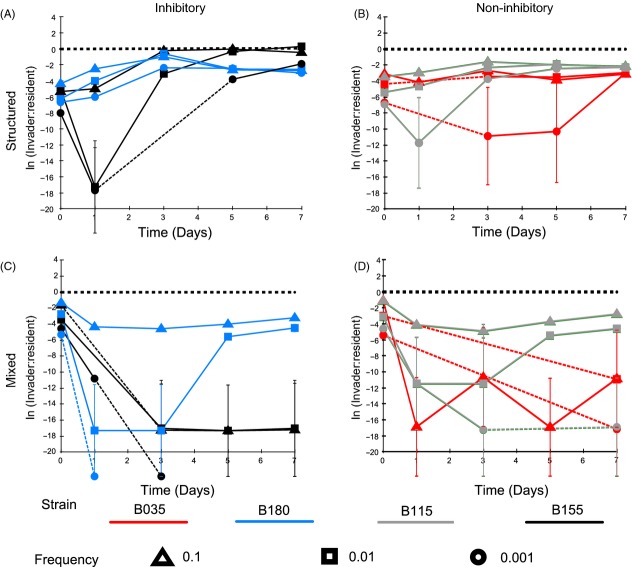
Toxin-producing (blue and black) and nonproducing (red and grey) isolates of *Staphylococcus epidermidis* invading populations of *S. aureus* (SH1000) at frequencies of 0.1 (triangle), 0.01 (square) and 0.001 (circle). Toxin-producing *S. epidermidis* isolates (155 and 180) and nonproducing *S. epidermidis* isolates (035 and 115) were introduced into a population of *S. aureus* (SH1000) at three different frequencies. This was carried under a spatially structured regime (A and B) and under a mixed regimen (C and D). The *x*-axis is the time in days, and the *y*-axis is the natural log of the invader to resident ratio. A dotted line in the time course shows when the population dipped below the experiment detection threshold (for clarity, these lines also cross the *x*-axis if the population went to extinction). There is a heavy dotted line at 0 on the *y*-axis to indicate an equal invader to resident ratio. The line crossing the *x*-axis symbolizes that the population went to extinction. Error bars represent the standard error of the mean (*n* = 3).

**Figure 3:**
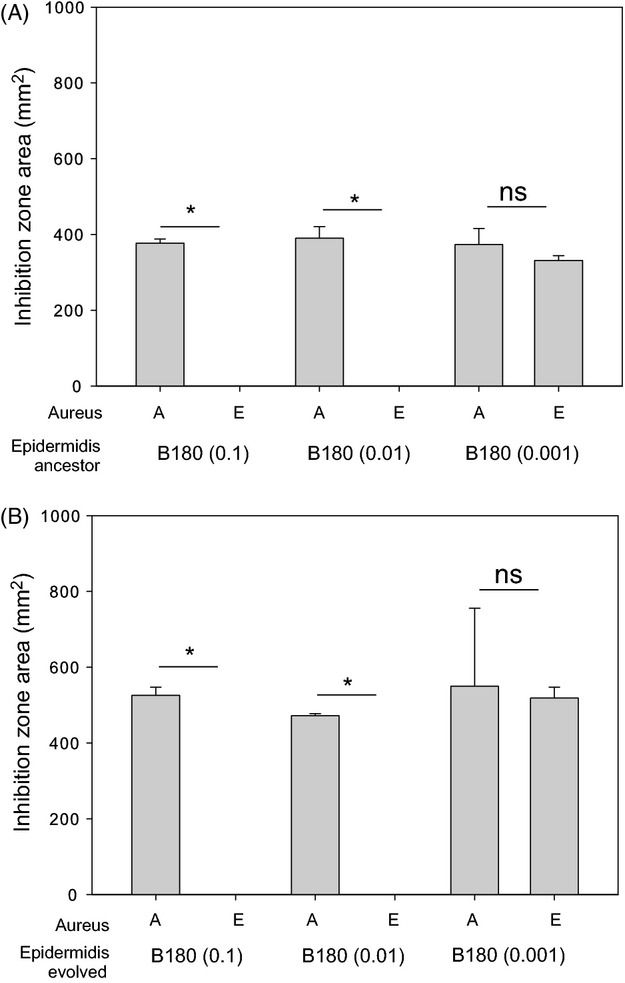
Resistance of evolved SH1000 resident after *Staphylococcus epidermidis* (B180) invasion. Panel A shows inhibition zone produced by the ancestral *S. epidermidis* strains, and panel B shows the inhibition zones produced by the evolved *S. epidermidis* strains. Both panels A and B show the inhibition zone area (mm^2^) produced by the toxin-producing *S. epidermidis* strains against the ancestral SH1000 (A) and the evolved SH1000 (E). Asterisks represent a significant difference between the inhibition zone areas of ancestral (A) and evolved (E) *S. aureus* strains as determined by a Fisher’s exact test. Each significance star represents a *P* value of 0.0022 which is significant when Bonferroni corrected for multiple comparisons with an alpha value of 0.1. Error bars represent the standard error of the mean.

Under structured conditions, the two invading, toxin-producing strains of *S. epidermidis* show different dynamics over time (Fig.2A). All starting frequencies of strain B155 increase after day 1 and approach a 1:1 invader to resident ratio, whereas strain B180 (starting frequencies 0.1 and 0.01) increases until day 3, after which they decrease. Spray assays were performed to test whether the decline in frequency of strain B180 populations (of starting frequency 0.1 and 0.01) was caused by resistance evolution in the resident *S. aureus* population. Ancestral and evolved resident *S. aureus* clones were sprayed over ancestral and evolved *S. epidermidis* strain B180 (Fig.3). These assays show that after 7 days, the resident *S. aureus* had evolved resistance to the invading *S. epidermidis* under structured conditions at starting frequencies of 0.1 and 0.01 (Fig.3) (Fisher’s exact test, *P* = 0.0022). Resistance was not seen in the *S. aureus* resident population when invaded with strain B180 at a starting frequency of 0.001 (Fig.3) (Fisher’s exact test, *P* = 1). Of note, evolved *S. epidermidis* strains (Fig.3B) produced larger inhibition zones against susceptible *S. aureus* than the ancestral *S. epidermidis* strains (Fig.3B) (paired *t*-test: *T* = 2.69, *P* = 0.03).

### Toxin-producing *S. epidermidis* strains resist invasion, especially in structured environments

**Figure 4:**
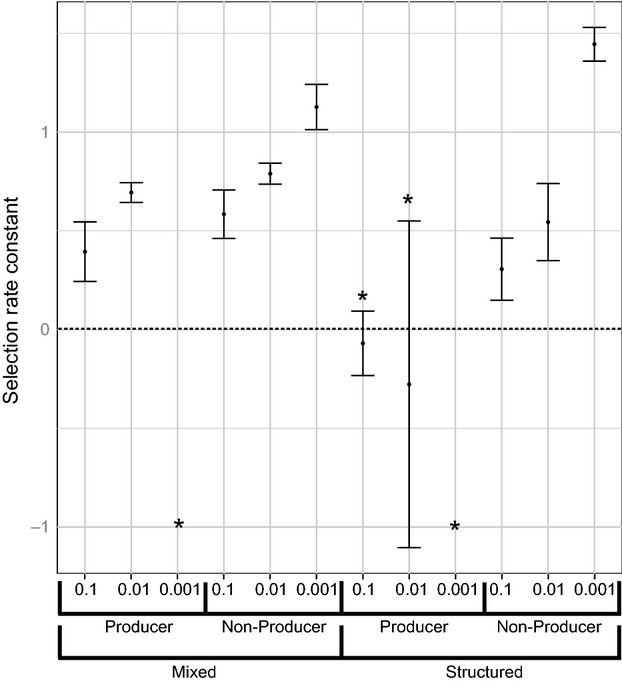
Selection rate coefficients for *Staphylococcus aureus* (SH1000) invading populations of *S. epidermidis*. *S. aureus* was introduced into populations of toxin-producing *S. epidermidis* isolates (155 and 180) and nonproducing isolates (035 and 115) at relative frequencies of 10, 100 and 1000. Each of the invasions was also carried out under a spatially structured treatment and a mixed treatment. Asterisks mark negative selection rate coefficients where invasion did not occur. Error bars represent the standard error of the mean.

**Figure 5:**
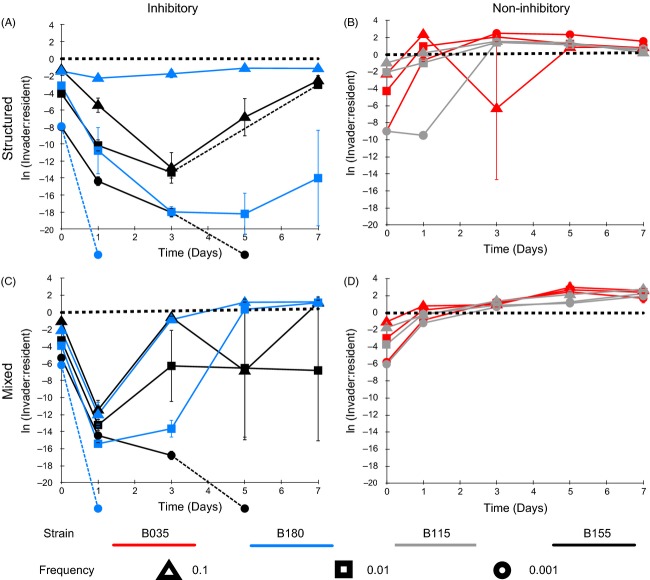
*Staphylococcus aureus* invading populations of toxin-producing (blue and black) and nonproducing (red and grey) *S. epidermidis* at frequencies of 0.1 (triangle), 0.01 (square) and 0.001 (circle). *S. aureus* strain (SH1000) was introduced into two different toxin-producing *S. epidermidis* populations (155 and 180), and two different nonproducing populations (035 and 115) at three different frequencies. This was carried under a spatially structured regime (A and B) and under a mixed regime (C and D). The *x*-axis is the time in days, and the *y*-axis is the natural log of the invader to resident ratio. A dotted line in the time course shows when the population dipped below the experiment detection threshold (for clarity, these lines also cross the *x*-axis if the population went to extinction). There is a heavy dotted line at 0 on the *y*-axis to indicate an equal invader to resident ratio. The line crossing the *x*-axis symbolizes that the population went to extinction. Error bars represent the standard error of the mean (*n* = 3).

Toxin-producing *S. epidermidis* strains were more resistant to invasion than nonproducing strains (inhibition, *F*_1,64_ = 124.95, *P* < 0.0001, Table4) and restricted invasion more effectively under structured environmental conditions (Figs4 and 5A) (structure × inhibition, *F*_1,64_ = 6.14, *P* < 0.05, Table4). Invasion of *S. aureus* into a toxin-producing *S. epidermidis* resident was positively frequency-dependent with highest initial frequencies invading the fastest and lower initial frequencies going to extinction (Fig.5A,C) (frequency × inhibition, *F*_1,64_ = 46.5, *P* < 0.001).

**Table 4:** Analysis of variance testing the main effects of successful invasion of *S. aureus* into populations of *S. epidermidis*. The table shows the results of a multifactorial anova. Both main effects and interactions are shown

	df	Sum sq	Mean sq	*F* value	*P* value
Frequency	1	2.721	2.721	8.1457	0.005810**
Structure	1	2.949	2.949	8.8266	0.004177**
Inhibition	1	41.744	41.744	124.9525	<2.2e-16***
Frequency × Structure	1	0.007	0.007	0.0198	0.88449
Frequency × Inhibition	1	15.554	15.554	46.5589	3.794e-09***
Structure × Inhibition	1	2.051	2.051	6.1382	0.015880*
Frequency × Structure × inhibition	1	0.398	0.398	1.1900	0.279418

df, Degrees of freedom; Sum sq, sum of squares; Mean sq, Mean of squares; *F* value, *F* statistic for terms in the row; *P* value, significance.

Asterisks indicate the significance levels at different thresholds. ^*^P < 0.05; ^**^P < 0.01, ^***^P < 0.001.

### Evolved resistance promotes *S. aureus* invasion

**Figure 6:**
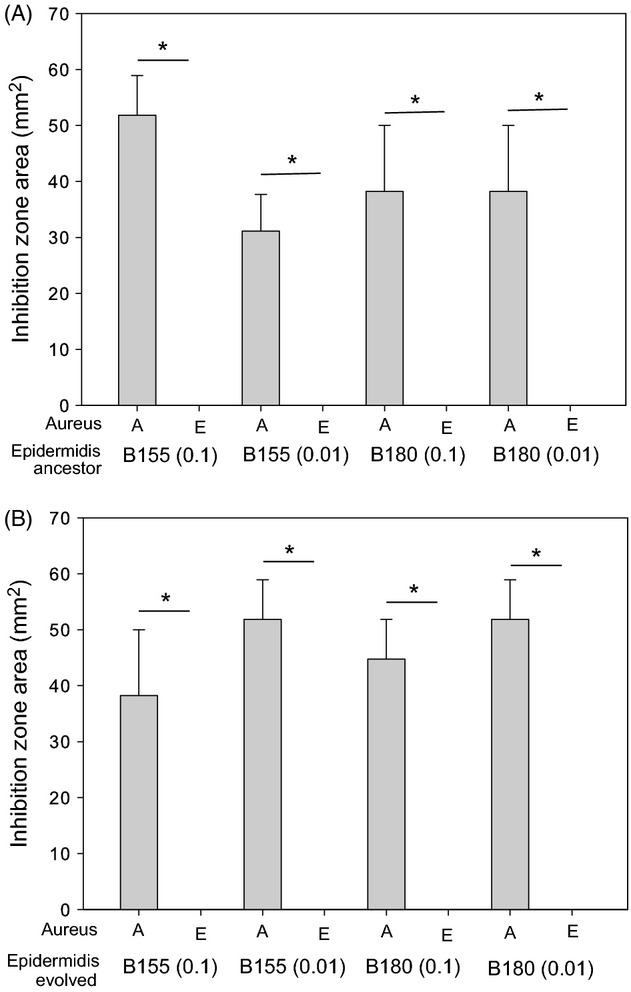
Resistance of evolved SH1000 after successful invasion. Panel A shows inhibition zone produced by the ancestral *Staphylococcus epidermidis* strains, and panel B shows the inhibition zones produced by the evolved *S. epidermidis* strains. Both panels A and B show the inhibition zone area (mm^2^) produced by the inhibitory *S. epidermidis* strains against the ancestral SH1000 (A) and the evolved SH1000 (E). The * represents a significant difference between the inhibition zone areas of ancestral (A) and evolved (E) *S. aureus* strains as determined by a Fisher’s exact test. Each significance star represents a *P* value of 0.0022, which is significant when Bonferroni corrected for multiple comparisons with an alpha value of 0.1. Error bars represent the standard error of the mean.

**Figure 7:**
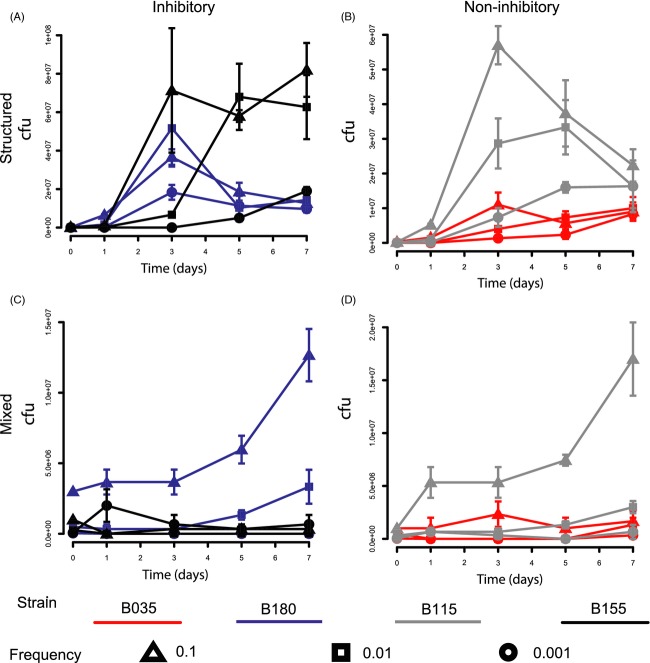
Toxin-producing (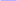 and 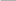) and nonproducing (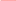
and 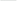) isolates of *Staphylococcus epidermidis* invading populations of *S. aureus* (SH1000) at frequencies of 0.1 (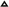), 0.01 (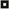) and 0.001 (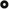). Toxin-producing *S. epidermidis* isolates (155 and 180) and nonproducing *S. epidermidis* isolates (035 and 115) were introduced into a population of *S. aureus* (SH1000) at three different frequencies. This was carried under a spatially structured regime (A and B) and under a mixed regimen (C and D). The *x*-axis is the time in days, and the *y*-axis is the colony-forming units (cfu) per plate. Error bars represent the standard error of the mean (*n* = 3).

**Figure 8:**
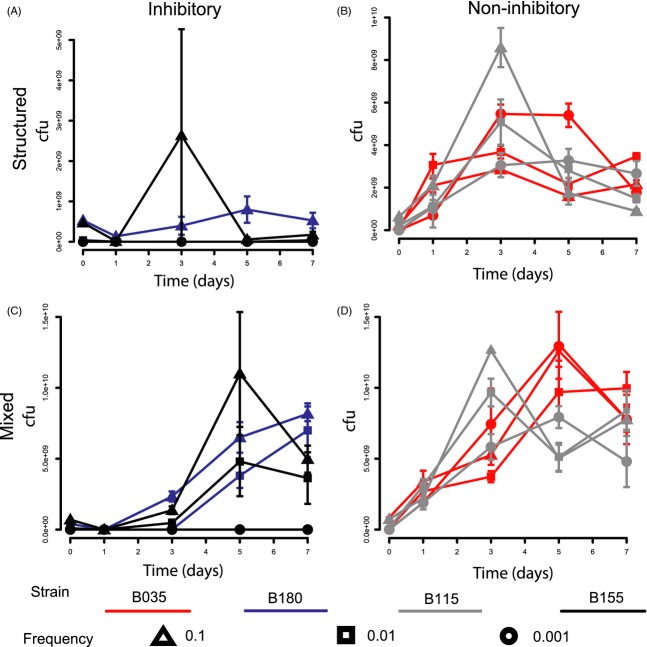
*Staphylococcus aureus* invading populations of toxin-producing (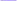 and 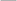) and nonproducing (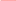 and 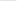) *S. epidermidis* at frequencies of 0.1 (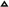), 0.01 (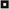) and 0.001 (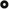). *S. aureus* strain (SH1000) was introduced into two different toxin-producing *S. epidermidis* populations (155 and 180), and two different nonproducing populations (035 and 115) at three different frequencies. This was carried under a spatially structured regime (A and B) and under a mixed regime (C and D). The *x*-axis is the time in days, and the *y*-axis is the colony-forming units (cfu) per plate. Error bars represent the standard error of the mean (*n* = 3).

*Staphylococcus aureus* was only able to invade toxin-producing *S. epidermidis* under mixed conditions (Fig.5C). To test whether the evolution of inhibitory toxin resistance by *S. aureus* was responsible for the invasion in a mixed environment (Figs4 and 5C), ancestral and evolved *S. aureus* strains were sprayed over ancestral and evolved *S. epidermidis* toxin-producing residents. In all cases, evolved *S. aureus* were resistant to the *S. epidermidis* toxin (Fig.6) (Fisher’s exact test, *P* = 0.0022).

## Discussion

We show that antimicrobial toxin production by *S. epidermidis* nasal isolates can have important effects on competition with *S. aureus*: interference competition acts both to promote invasion by, and to prevent invasion into, *S. epidermidis* populations. This supports the growing body of evidence that species interactions can play an important role determining species distributions in nasal microbial communities (Lina et al. 2003; Frank et al. 2010; Wos-Oxley et al. 2010; Yan et al. 2013), and more specifically that trait variation, in this case in the toxins mediating interference competition, could act to prevent *S. aureus* nasal carriage (Libberton et al. 2014).

Our findings highlight the critical role for spatial structure in determining the outcome of interference competition. If spatial structure is maintained, then inhibitor-producing bacteria can better prevent the invasion-from-rare of *S. aureus*, whereas unstructured environments generally do not favour the production of inhibitory toxins. Spatial structure is likely to be an important component of life in the anterior nares. While nutrient agar is clearly not equivalent to this environment and transfers using velvet may select those bacteria growing nearest to the colony surface, our manipulation of spatial structure is arguably more relevant to the nasal environment than comparing agar plates to liquid culture (Chao and Levin 1981). The macrotopography of the nares is irregular, with ridges and recesses providing spatially discrete surfaces. The base layer of the nares is comprised of a squamous epithelium that microbes colonize and form spatially discrete groups (Uraih and Maronpot 1990; Yuki et al. 2000; Dongari-Bagtzoglou et al. 2009). Spatial population structure is therefore expected to be present, but is likely to be disrupted by changes to the squamous epithelium, the flow of air (Churchill et al. 2004) and mucus (Proctor et al. 1973) through the nasal passages and mechanical disruptions (e.g. nose picking). Factors that reduce the spatial structuring in nasal communities could weaken the ability of inhibitory resident species to prevent invasion by *S. aureus*.

Further, our data demonstrate that rapid evolution of resistance to antimicrobial toxins can determine the outcome of interference competition. Positive frequency-dependent fitness was observed for *S. aureus* invading inhibitory residents. This may have occurred due to a protective effect from a lager inoculum neutralizing the effect of the toxin. This is unlikely, however, as protection caused by large bacterial densities is typically a result of quenching and lowering the local concentration of available toxin. In this experimental set-up, toxin would be produced during growth phases every time the population is transferred, which would overcome any possible quenching effect. The more likely explanation for resistance evolution is that the higher inoculation frequencies increased the chance of these invading populations containing beneficial resistance mutations. This outcome is similar to theory predicting conditions for adaptation to black hole sink environments where an increased immigration rate (i.e., increased frequency of the invading population) increases the probability of adaptation (Holt and Gaines 1992; Perron et al. 2008). Moreover, this scenario suggests that an inhibitor-producing community could resist invasion from rare by *S. aureus*, because of a low probability of resistance evolution. When toxin-producing *S. epidermidis* were invading resident *S. aureus,* evolution of resistance in *S. aureus* resident populations was most likely when the toxin-producing invaders were relatively common (Fig.3). Higher frequencies of invading toxin producers would have produced more of the toxin, generating stronger selection for resistance; additionally, a larger fraction of the *S. aureus* populations would have been exposed to these toxins. This relationship suggests that resistance evolution by residents may frequently impede invasion by toxin-producing strains, because resident populations are unlikely to be mutation-limited and selection for resistance progressively strengthens as an invasion proceeds. Intriguingly, in spatially structured environments, the evolved invading *S. epidermidis* (B180) showed greater inhibitory activity on ancestral SH1000 than the ancestral B180 genotype (Fig.3). This suggests that the toxin producer coevolved to meet the survival challenges posed by increasingly resistant *S. aureus* populations. The evolved *S. epidermidis* may have upregulated production of the inhibitory toxin, or alternatively initiated production of alternative toxins. However, in the absence of knowledge of the mechanism of inhibition, this remains unclear.

One strength of this study is the use of toxin-producing strains isolated from the nares of healthy volunteers and not isogenic toxin-producing and nonproducing laboratory strains. Although nasal isolates are more difficult to compare with well-characterized laboratory strains, they have greater relevance to future development of therapeutic strategies and provide added realism to laboratory models of colonization. There are also other limitations, for example the zones of inhibition produced from B180 and B155 had different areas, which implies differential expression of the same toxin or discrete toxins. Resource competition and adaptation to the growth medium could affect the outcomes described and contribute to the interactions between the pairs of bacteria, but these aspects would require further study to describe.

It is stated that preventing *S. aureus* carriage significantly reduces the risk of infection (Von Eiff et al. 2001). Our findings support the possibility that manipulation of the microbial community in the human nose to increase the frequency of inhibitor-producing residents could reduce *S. aureus* colonization. The human gut has been a model system for therapeutic manipulation of the microbial flora for many years (Borody et al. 1989; Landy et al. 2011). If the models of colonization and their outcomes can be replicated in the nasal environment, it would represent a novel way to limit *S. aureus* carriage that is correlated with associated life-threatening infections.

## Data archiving statement

Data available from the Dryad Digital Repository: http://dx.doi.org/10.5061/dryad.nb535.
